# Serotonin-mediated modulation of Na^+^/K^+ ^pump current in rat hippocampal CA1 pyramidal neurons

**DOI:** 10.1186/1471-2202-13-10

**Published:** 2012-01-19

**Authors:** Li Nan Zhang, Su Wen Su, Fang Guo, Hui Cai Guo, Xiao Lu Shi, Wen Ya Li, Xu Liu, Yong Li Wang

**Affiliations:** 1 Department of Pharmacology, Hebei Medical University, 361 Zhongshan East Road, Shijiazhuang, 050017, China; 2 Department of Pharmacy, College of Chemical and Pharmaceutical Engineering, Hebei University of Science and Technology, 70 Yuhua East Road, Shijiazhuang, 050018, China; 3 Department of Toxicology, Hebei Medical University, 361 Zhongshan East Road, Shijiazhuang, 050017, China

## Abstract

**Background:**

The aim of this study was to investigate whether serotonin (5-hydroxytryptamine, 5-HT) can modulate Na^+^/K^+ ^pump in rat hippocampal CA1 pyramidal neurons.

**Results:**

5-HT (0.1, 1 mM) showed Na^+^/K^+ ^pump current (Ip) densities of 0.40 ± 0.04, 0.34 ± 0.03 pA/pF contrast to 0.63 ± 0.04 pA/pF of the control of 0.5 mM strophanthidin (Str), demonstrating 5-HT-induced inhibition of Ip in a dose-dependent manner in hippocampal CA1 pyramidal neurons. The effect was partly attenuated by ondasetron, a 5-HT_3 _receptor (5-HT_3_R) antagonist, not by WAY100635, a 5-HT_1A_R antagonist, while 1-(3-Chlorophenyl) biguanide hydrochloride (m-CPBG), a 5-HT_3_R specific agonist, mimicked the effect of 5-HT on Ip.

**Conclusion:**

5-HT inhibits neuronal Na^+^/K^+ ^pump activity via 5-HT_3_R in rat hippocampal CA1 pyramidal neurons. This discloses novel mechanisms for the function of 5-HT in learning and memory, which may be a useful target to benefit these patients with cognitive disorder.

## Background

5-HT, as a neurotransmitter or neuromodulator in the central nervous system, plays a critical role in the control of blood pressure, body temperature, sleep, depression, anxiety, epilepsy [1-4]. Additionally, the modulation of the serotonergic system affects long-term potentiation (LTP) and long-term depression (LTD), the likely neurophysiologic derivates of learning and memory formation, which has been involved in the treatment of Alzheimer's disease [5-8]. Some studies demonstrate that 5-HT_1A_R-knockout animals show a deficit in hippocampal-dependent learning and memory, such as the hidden platform (spatial) version of the Morris water maze and the delayed version of the Y maze [9], while the stimulation of 5-HT_1A_R mediates enhancement of LTP [10] and prevents the impairment of learning and memory [11,12]. Therefore, the stimulation of 5-HT_1A_R may be useful in the symptomatic treatment of human memory disturbances. However, accumulated clinical reports support that the injection of 5-HT_3_R antagonists facilitates the induction of LTP, and enhances the retention and consolidation of memory in hippocampal dependent tasks [13-15]. These clinical application of 5-HT_3_R antagonists have been found to improve memory in schizophrenic or Alzheimer demented patients [16,17]. Therefore, 5-HT_3_R also plays a critical role in cognitive function.

In addition to increasing neuronal excitability [18], inhibition of Na^+^/K^+ ^pump activity can induce LTD whereas depotentiate LTP [19], and then cause impairment of learning and memory and amnesia [20,21]. Herein, in the present study, we investigate if a relationship occurs between 5-HT and Na^+^/K^+ ^pump in hippocampal CA1 pyramidal neurons, which may provide new insights in the mechanisms responsible for the 5-HT-mediated modulation of learning and memory.

## Results and discussion

### 5-HT-mediated inhibition of Ip in rat hippocampal CA1 pyramidal neurons

0.5 mM Str often did not recover completely in hippocampal CA1 slices even after prolonged washout in the present study, consistent with the previous study that Na^+^/K^+ ^pump inhibition by Str was effectively irreversible [22]. Furthermore, 10 μM Str did not recover completely in rat ventral midbrain slices [23]. Therefore, Str perfusion was applied one time in one brain slice.

**Figure 1:**
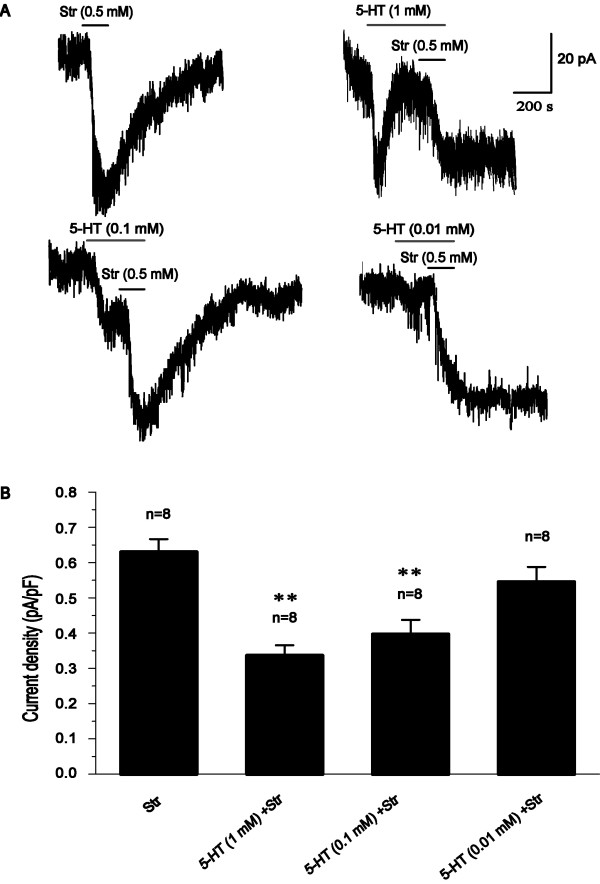
**5-HT-mediated inhibiton of Ip in rat hippocampal CA1 pyramidal neurons**. (A) The representative tracings of 5-HT (0.01-1 mM)-mediated effect of Ip contrast with the control of Str-mediated Ip. (B) 5-HT at 0.1 and 1 mM significantly mediated concentration-dependent suppression of Ip contrast with the control of Str (0.5 mM) (**P < 0.01). The number of all cells tested is indicated in each column.

In the present study, Ip densities affected by 5-HT (0.1, 1 mM) were 0.40 ± 0.04, 0.34 ± 0.03 pA/pF contrast to 0.63 ± 0.04 pA/pF of the control of 0.5 mM Str (Figure 1), demonstrating 5-HT inhibits Na^+^/K^+ ^pump activity in hippocampal CA1 pyramidal neurons. Some studies have reported that 5-HT inhibits Na^+^/K^+ ^pump in T sensory neurons of the leech [24] and kidney [25], then depresses the after-hyperpolarization. However, other studies have showed that 5-HT activates glial Na^+^/K^+ ^pump activity in rat cerebral cortex and hippocampus [26,27]. 5-HT stimulated synaptic membrane Na^+^/K^+ ^pump from the rabbit cerebrum, but did not influence the activity of this enzyme in the other brain regions [28]. These studies suggested that the regulation of 5-HT-induced Na^+^/K^+ ^pump activity may be attributable to tissue and cell specificity.

Furthermore, some studies reported that the application of 1 μM 5-HT prevented depotentiation but not LTP induced by high-frequency stimulation, whereas bath application of 100 μM 5-HT blocked the induction of tetanus-induced LTP [29], consistent with the previous study that 5-HT (30 μM) prevented LTP induced by a primed burst in rat hippocampal CA1 region [30]. Accordingly, different concentrations of 5-HT may have different modulation of learning and memory. Moreover, the inhibition of Na^+^/K^+ ^pump activity can induce LTD whereas depotentiate LTP [19], and then cause impairment of learning and memory and amnesia [20,21]. Herein, in the present study, 5-HT-mediated inhibition of Na^+^/K^+ ^pump activity may disclose novel mechanisms in learning and memory. Further studies should be done to explore the mechanism.

### 5-HT mediated inhibiton of Ip via 5-HT_3_R not 5-HT_1A_R

**Figure 2:**
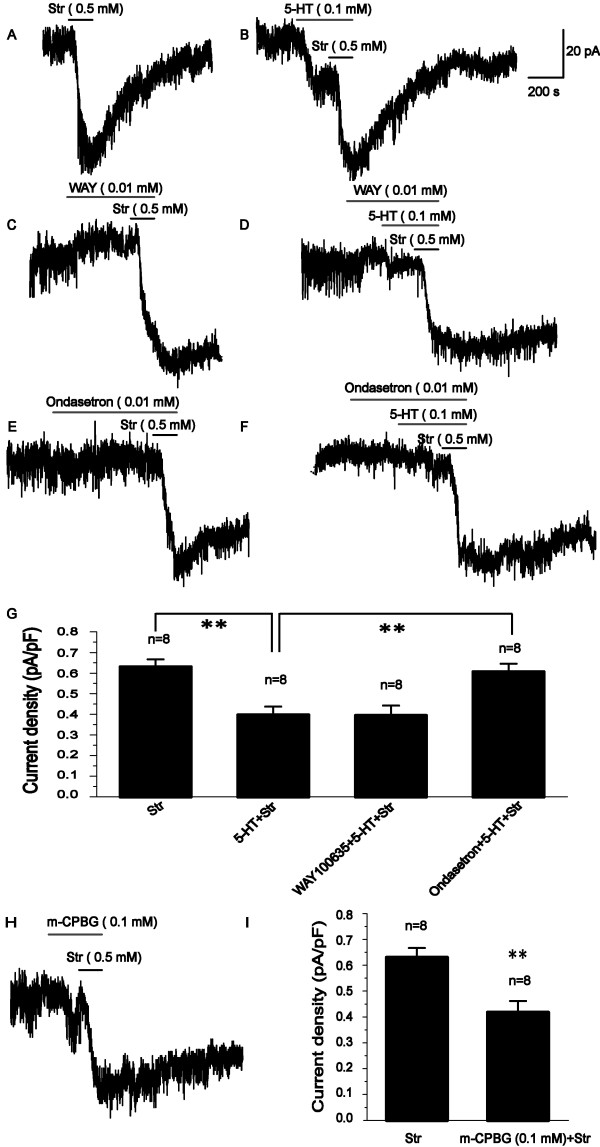
**5-HT-mediated inhibiton of Ip is mediated by 5-HT_3_R, but not by 5-HT_1A_R in hippocampus**. (A) The representative tracing of 0.5 mM Str- mediated Ip in the control. (B) The representative tracing of 5-HT (0.1 mM) -mediated inhibiton of Ip. WAY100635, a 5-HT_1A_R antagonist, alone did not affect Ip (P > 0.05) (C), and did not block 5-HT-mediated inhibiton of Ip (D). Ondasetron, a 5-HT_3_R antagonist, alone did not affect Ip (P > 0.05) (E), whereas attenuated 5-HT-mediated inhibiton of Ip (F). (G) 5-HT-mediated inhibiton of Ip is blocked by ondasetron, but not WAY100635. (H) The representative tracing of m-CPBG (0.1 mM)-mediated inhibiton of Ip. (I) m-CPBG (0.1 mM) -mediated inhibiton of Ip. Values significantly different by Student's t-test from results are indicated as **P < 0.01.

To identify the specific 5-HTR involved in the regulation of Ip, we focused on the 5-HT_1A_R and 5-HT_3_R that are abundant in all hippocampal layers and subregions [31-33]. In the present study, "WAY1000635 (a 5-HT_1A_R antagonist) + 5-HT + Str" treatment yielded the similar result as "5-HT + Str" treatment (Figure 2), i.e., the application of the antagonist for 5-HT_1A_R had no effect on 5-HT-mediated inhibition of Ip, suggesting that 5-HT_1A_R was not involved in 5-HT-mediated inhibition of Ip. Some studies have reported that 5-HT_1A_R mediates enhancement of LTP in hippocampal dentate gyrus [10] and prevents the impairment of learning and memory [11,12], whereas inhibits LTP in the hippocampal CA1 field and visual cortex [34,35], demonstrating the different effects of 5-HT_1A_R on synaptic transmission in different tissues. Therefore, there still are some arguments about 5-HT_1A_R-induced-modulation of LTP. In the present study, 5-HT_1A_R did not antagonize 5-HT-mediated inhibition of Ip, demonstrating that Na^+^/K^+ ^pump may be not involved in 5-HT_1A_R-mediated modulation of memory.

In the presence of ondasetron, a 5-HT_3_R antagonist, 5-HT-mediated inhibition of Ip was blocked from 0.40 ± 0.04 to 0.61 ± 0.04 pA/pF (Figure 2), while m-CPBG, a 5-HT_3_R specific agonist, mimicked the effect of 5-HT on Ip (Figure 2). These results show, for the first time, that the inhibition of 5-HT-mediated Ip is primarily mediated by 5-HT_3_R in hippocampal CA1 pyramidal neurons. On the subcellular level, both presynaptic and postsynaptic 5-HT_3_R can be found. Presynaptic 5-HT_3_R is involved in mediating or modulating neurotransmitter release. Postsynaptic 5-HT_3_R is preferentially expressed on interneurons [36,37], and there is also 5-HT_3_R in postsynaptic pyramidal neurons [38-42]. For example, electrophysiological studies in postsynaptic pyramidal neurons in the hippocampal CAl region or hippocampal primary cultures showed the activation of 5-HT_3_R [38-42]. Furthermore, 5-HT_3_R is a unique serotonin receptor as it acts as a ligand-gated ion channel, whereas all the other types of serotonin receptors belong to the G protein-coupled receptor superfamily, which may be the reason of 5-HT_3_R, rather than 5-HT_1A_R, is the relevant 5-HTR for 5-HT-mediated inhibition of Ip in the present study. This still deserves further investigations.

Some studies indicate that overexpression of the 5-HT_3_R in mouse forebrain results in enhanced hippocampal-dependent learning and attention involved in fear conditioning [43], whereas most reports show that 5-HT_3_R antagonists can facilitate LTP and enhance the retention and consolidation of memory in hippocampal dependent tasks [13]. Furthermore, the complete abolishment of 5-HT innervation in the hippocampus increases LTP in vivo [44]-which would suggest that, on balance, 5-HT may exert a negative influence on LTP via 5-HT_3_R and then impair learning and memory [14,15]. Clinically application of 5-HT_3_R antagonists have been found to improve memory in schizophrenic or Alzheimer demented patients [16,17]. It is, however, not clear whether this effect is specific to LTP, or secondary to other changes.

Some studies reported that inhibition of Na^+^/K^+ ^pump activity can induce LTD whereas depotentiate LTP [19], and then cause impairment of learning and memory and amnesia [19-21,45-47]. Moreover, The initial stationary phase of the LTP was followed by a decrease in Na^+^/K^+ ^pump activity of neurons and an augmentation of Na^+^/K^+ ^pump activity in the glial cells [48]. These studies supported that there may be some relationship between Na^+^/K^+ ^pump and LTP. The present results show that 5-HT can suppress Ip in hippocampal CA1 pyramidal neurons via 5-HT_3_R, consistent with the previous study that 5-HT_3_R partly mediated the decrease of Na^+^/K^+ ^pump activity induced by cocaine in neuronal-like cells [49], suggesting that inhibition of Na^+^/K^+ ^pump activity may be involved in 5-HT_3_R-induced modulation of learning and memory. This provides new insights for the possible synaptic role of 5-HT via 5-HT_3_R in cognitive function and neuronal development through Na^+^/K^+ ^pump, which may be a useful target to benefit these patients with cognitive disorder.

## Conclusion

5-HT inhibits neuronal Na^+^/K^+ ^pump activity via 5-HT_3_R in hippocampal CA1 pyramidal neurons, which may disclose novel mechanisms for the function of 5-HT in learning and memory.

## Methods

### Solutions and chemicals

Str, ondasetron and WAY-100635 were purchased from Sigma (St. Louis, MO, USA). 5-HT and tetrodotoxin (TTX) were purchased from Alexis (San Diego, CA, USA). m-CPBG and other chemicals were purchased from Alfa Aesar (Ward Hill, MA, USA). Str was dissolved in DMSO and further diluted 1: 1000 in artificial cerebrospinal fluid (ACSF) containing (mM): NaCl 119, KCl 5.4, MgCl_2 _1.3, NaH_2_PO_4_•2H_2_O 1, D-Glucose 11, NaHCO_3 _26.2, CaCl_2 _2.5. Control solutions of 1: 1000 DMSO had no effect on membrane current. TTX was dissolved in dilute acetic acid (PH 4.8-4.9). 5-HT, ondasetron, m-CPBG and WAY-100635 were dissolved in sterile water and stored as stock solutions. All stock solutions were stored as frozen aliquots at -20°C.

### Brain hippocampal slice preparation and loading

Sprague-Dawley rats of 12-14 days were deeply anesthetized with sodium pentobarbital (45 mg kg^-1^, i.p.) and then rapidly decapitated. Our experiments were approved by Animal Care Committee of Hebei Medical University. Appropriate experimental procedures were taken to minimize pain or discomfort. The brain was quickly removed from the skull and transverse hippocampal slices (300 μm thick) were obtained by cutting with a vibroslice MA752 (Campden Instruments, Loughborough, UK) in ice-cold ACSF well-saturated with 95% O_2 _and 5% CO_2 _(PH 7.3-7.4). These slices were pre-incubated in oxygenated ACSF at room temperature (22-25°C) for 1 h.

### Electrophysiological recordings of Na^+^, K^+^-pump currents

**Figure 3:**
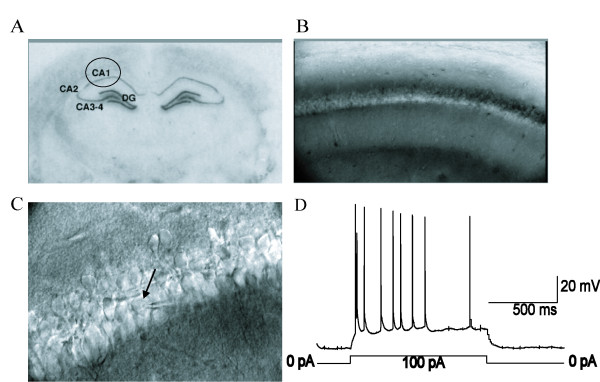
**The location visualization and electrophysiological characteristics of rat hippocampal CA1 pyramidal neurons**. A. Schematic representation of the location of the hippocampal CA1 slice. B. The hippocampal CA1 location as visualized with ×10 infrared video microscopy. C. The hippocampal CA1 pyramidal neurons as visualized with ×40 water immersion lens of infrared video microscopy. Patch pipette is visible during whole-cell recording from the recorded neuron on right. D. Action potentials of the hippocampal CA1 pyramidal neuron during a depolarizing current pulse from the resting potential of -60 mV. Note the spike frequency adaptation. The whole cell recording under the current clamp method was used. The amplitude and the time of injected currents were shown on top of membrane potential trace.

**Figure 4:**
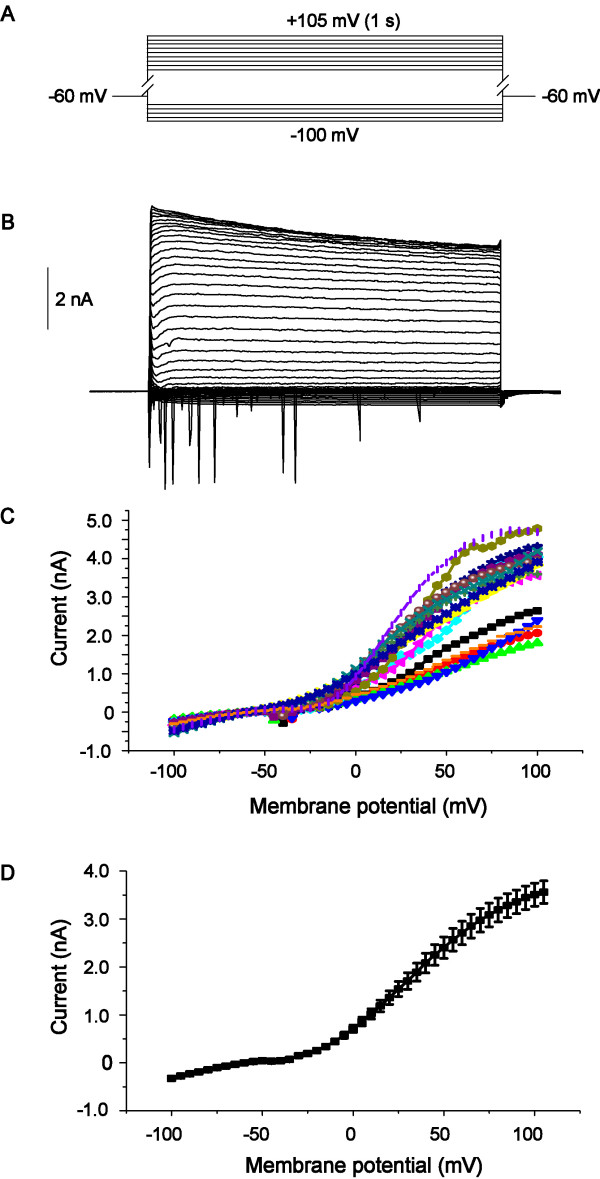
**I-V curve of recorded neurons**. A, Schematic representation of the protocol of the recorded neurons; B, Representative current traces recorded from a typical hippocampal CA1 pyramidal neuron. Cell was held at -60 mV and stepped from -100 mV to +105 mV in 5 mV interval for 1-s duration, followed by a step to -60 mV once. C and D, I-V plots were constructed from the values of traces.

The hippocampal slice containing CA1 pyramidal neurons was transferred to a submerged recording chamber and continuously superfused with oxygenated ACSF (containing 2 mM BaCl_2_, 0.2 mM CdCl_2 _and 0.5 μM TTX) at a rate of 2 ml•min^-1 ^at room temperature [50]. Only one cell was measured from each brain slice. Hippocampal CA1 pyramidal neurons were visualized by their location [51,52] using infrared differential interference contrast video microscopy and a ×40 water immersion lens (Zeiss Axioskop), as shown in Figure 3A, B, C. In addition to the discrimination by the location, we determined pyramidal neurons by electrophysiological characteristics. We used current-clamp method to record the action potentials of hippocampal CA1 pyramidal neurons held at 0 pA and elicited the action potential by current injection for 1 s. Hippocampal CA1 pyramidal neurons, which were recorded with an EPC-10 amplifier (HEKA Instruments), usually exhibited spike frequency adaptation in response to a depolarizing current pulse in whole-cell current-clamp recordings, as shown in Figure 3D, which is a common characteristic of pyramidal neurons [53,54]. Patch clamp electrodes with resistances of 4-6 MΩ were made by a horizontal puller (Model P-97, Suttter Instruments), and they were filled with the pipette solution containing (mM): Gluconatic acid 125, TEACl 10, CsOH 125, MgCl_2 _2, NaCl 8, HEPES 10, EGTA 0.2, Na_2_ATP 3, Na_2_GTP 0.3 (PH 7.2). Currents were digitally sampled at 100 μs (10 kHz) and filtered at 2.9 kHz by a Bessel filter. The layer II/III hippocampal CA1 pyramidal neurons in acute hippocampal slices (300 μm thick) were held at -60 mV, at which holding potential, the membrane patch was most stable in the present experimental conditions. The average resting membrane potential was -65 ± 8.6 mV (mean ± SD, n = 105 cells), with the average input resistance of 117.3 ± 28.5 MΩ (mean ± SD, n = 93 cells). The input resistance of the cell membrane was derived by calculating the reciprocal of the slope of the I-V curve at the zero-current potential (Figure 4). To isolate Ip in hippocampal CA1 pyramidal neurons during stable recordings, Str (0.5 mM) was used to inhibit the Na^+^/K^+ ^pump, and an inward move of holding current generated. To detect the effect of 5-HT on Ip, 5-HT was given for 3-4 min, then Str was co-applied with 5-HT, and the produced inward current was contrasted with the control Ip of Str [50].

### Statistical analysis

Results were expressed as the mean ± S.E.M., and n indicated the number of slices studied. Statistical comparisons were made using the Student's t-test for unpaired samples, and significant differences were defined as having a P-value less than 0.05.

## Authors' contributions

LNZ, SWS, FG and YLW conceived and designed the study. LNZ, HCG, XLS and YLW coordinated and performed the experiments. LNZ, XL and WYL performed data analysis. LNZ and YLW interpreted the results and wrote the paper. All authors read and approved the final manuscript.

## References

[B1] Villalon CM Centurion D Bravo G De Vries P Saxena PR Ortiz MI Further pharmacological analysis of the orphan 5-HT receptors mediating feline vasodepressor responses: close resemblance to the 5-HT7, receptor Naunyn Schmiedebergs Arch Pharmacol 2000 361 665 671 10.1007/s002100000256 10882042

[B2] Rausch JL Johnson ME Corley KM Hobby HM Shendarkar N Fei Y Ganapathy V Leibach FH Depressed patients have higher body temperature: 5-HT transporter long promoter region effects Neuropsychobiology 2003 47 120 127 10.1159/000070579 12759553

[B3] Morrow JD Vikraman S Imeri L Opp MR Effects of serotonergic activation by 5-hydroxytryptophan on sleep and body temperature of C57BL/6J and interleukin-6-deficient mice are dose and time related Sleep 2008 31 21 33 18220075 10.1093/sleep/31.1.21 PMC2225553

[B4] Bagdy G Kecskemeti V Riba P Jakus R Serotonin and epilepsy J Neurochem 2007 100 857 873 10.1111/j.1471-4159.2006.04277.x 17212700

[B5] Huang YY Kandel ER 5-Hydroxytryptamine induces a protein kinase A/mitogen-activated protein kinase-mediated and macromolecular synthesis-dependent late phase of long-term potentiation in the amygdala J Neurosci 2007 27 3111 3119 10.1523/JNEUROSCI.3908-06.2007 17376972 PMC6672482

[B6] Mansilla-Olivares A [Signal transduction, pillar of the neurobiological integration of memory. An alternative view to the cholinergic hypothesis] Gac Med Mex 2005 141 513 526 16381507

[B7] Inaba M Maruyama T Yoshimura Y Hosoi H Komatsu Y Facilitation of low-frequency stimulation-induced long-term potentiation by endogenous noradrenaline and serotonin in developing rat visual cortex Neurosci Res 2009 64 191 198 10.1016/j.neures.2009.02.014 19428700

[B8] Nitsche MA Kuo MF Karrasch R Wachter B Liebetanz D Paulus W Serotonin affects transcranial direct current-induced neuroplasticity in humans Biol Psychiatry 2009 66 503 508 10.1016/j.biopsych.2009.03.022 19427633

[B9] Sarnyai Z Sibille EL Pavlides C Fenster RJ McEwen BS Toth M Impaired hippocampal-dependent learning and functional abnormalities in the hippocampus in mice lacking serotonin(1A) receptors Proc Natl Acad Sci USA 2000 97 14731 14736 10.1073/pnas.97.26.14731 11121072 PMC18987

[B10] Sanberg CD Jones FL Do VH Dieguez D Jr Derrick BE 5-HT1a receptor antagonists block perforant path-dentate LTP induced in novel, but not familiar, environments Learn Mem 2006 13 52 62 10.1101/lm.126306 16452654 PMC1360133

[B11] Carli M Balducci C Samanin R Low doses of 8-OH-DPAT prevent the impairment of spatial learning caused by intrahippocampal scopolamine through 5-HT(1A) receptors in the dorsal raphe Br J Pharmacol 2000 131 375 381 10.1038/sj.bjp.0703567 10991934 PMC1572321

[B12] Meeter M Talamini L Schmitt JA Riedel WJ Effects of 5-HT on memory and the hippocampus: model and data Neuropsychopharmacology 2006 31 712 720 10.1038/sj.npp.1300869 16132065

[B13] Staubli U Xu FB Effects of 5-HT3 receptor antagonism on hippocampal theta rhythm, memory, and LTP induction in the freely moving rat J Neurosci 1995 15 2445 2452 7891179 10.1523/JNEUROSCI.15-03-02445.1995 PMC6578162

[B14] Meneses A A pharmacological analysis of an associative learning task: 5-HT(1) to 5-HT(7) receptor subtypes function on a pavlovian/instrumental autoshaped memory Learn Mem 2003 10 363 372 10.1101/lm.60503 14557609 PMC218002

[B15] Hong E Meneses A Systemic injection of p-chloroamphetamine eliminates the effect of the 5-HT3 compounds on learning Pharmacol Biochem Behav 1996 53 765 769 10.1016/0091-3057(95)02104-3 8801576

[B16] Levkovitz Y Arnest G Mendlovic S Treves I Fennig S The effect of Ondansetron on memory in schizophrenic patients Brain Res Bull 2005 65 291 295 10.1016/j.brainresbull.2003.09.022 15811593

[B17] Dysken M Kuskowski M Love S Ondansetron in the treatment of cognitive decline in Alzheimer dementia Am J Geriatr Psychiatry 2002 10 212 215 11925283

[B18] McCarren M Alger BE Sodium-potassium pump inhibitors increase neuronal excitability in the rat hippocampal slice: role of a Ca2+-dependent conductance J Neurophysiol 1987 57 496 509 2435860 10.1152/jn.1987.57.2.496

[B19] Reich CG Mason SE Alger BE Novel form of LTD induced by transient, partial inhibition of the Na, K-pump in rat hippocampal CA1 cells J Neurophysiol 2004 91 239 247 14715719 10.1152/jn.00722.2003

[B20] Moseley AE Williams MT Schaefer TL Bohanan CS Neumann JC Behbehani MM Vorhees CV Lingrel JB Deficiency in Na, K-ATPase alpha isoform genes alters spatial learning, motor activity, and anxiety in mice J Neurosci 2007 27 616 626 10.1523/JNEUROSCI.4464-06.2007 17234593 PMC6672804

[B21] Sato T Tanaka K Ohnishi Y Teramoto T Irifune M Nishikawa T Effects of steroid hormones on (Na+, K+)-ATPase activity inhibition-induced amnesia on the step-through passive avoidance task in gonadectomized mice Pharmacol Res 2004 49 151 159 10.1016/j.phrs.2003.09.006 14643695

[B22] Baker PF Willis JS Inhibition of the sodium pump in squid giant axons by cardiac glycosides: dependence on extracellular ions and metabolism J Physiol 1972 224 463 475 5071403 10.1113/jphysiol.1972.sp009905 PMC1331500

[B23] Shen KZ Johnson SW Sodium pump evokes high density pump currents in rat midbrain dopamine neurons J Physiol 1998 512 Pt 2 449 457 9763634 10.1111/j.1469-7793.1998.449be.x PMC2231210

[B24] Catarsi S Brunelli M Serotonin depresses the after-hyperpolarization through the inhibition of the Na+/K+ electrogenic pump in T sensory neurones of the leech J Exp Biol 1991 155 261 273 1849955 10.1242/jeb.155.1.261

[B25] Stepp LR Novakoski MA Effect of 5-hydroxytryptamine on sodium- and potassium-dependent adenosine triphosphatase and its reactivity toward ouabain Arch Biochem Biophys 1997 337 43 53 10.1006/abbi.1996.9762 8990266

[B26] Pena-Rangel MT Mercado R Hernandez-Rodriguez J Regulation of glial Na+/K+-ATPase by serotonin: identification of participating receptors Neurochem Res 1999 24 643 649 10.1023/A:1021048308232 10344593

[B27] Mercado R Hernandez J Regulatory role of a neurotransmitter (5-HT) on glial Na+/K(+)-ATPase in the rat brain Neurochem Int 1992 21 119 127 10.1016/0197-0186(92)90074-2 1303137

[B28] Logan JG O'Donovan DJ The effects of ouabain and the activation of neural membrane ATPase by biogenic amines J Neurochem 1976 27 185 189 10.1111/j.1471-4159.1976.tb01562.x 134132

[B29] Normann C Clark K Selective modulation of Ca(2+) influx pathways by 5-HT regulates synaptic long-term plasticity in the hippocampus Brain Res 2005 1037 187 193 10.1016/j.brainres.2005.01.001 15777768

[B30] Corradetti R Ballerini L Pugliese AM Pepeu G Serotonin blocks the long-term potentiation induced by primed burst stimulation in the CA1 region of rat hippocampal slices Neuroscience 1992 46 511 518 10.1016/0306-4522(92)90140-W 1545909

[B31] Burnet PW Eastwood SL Lacey K Harrison PJ The distribution of 5-HT1A and 5-HT2A receptor mRNA in human brain Brain Res 1995 676 157 168 10.1016/0006-8993(95)00104-X 7796165

[B32] Parker RM Barnes JM Ge J Barber PC Barnes NM Autoradiographic distribution of [3H]-(S)-zacopride-labelled 5-HT3 receptors in human brain Journal of the Neurological Sciences 1996 144 199 127 10.1016/s0022-510x(96)00211-0 8994113

[B33] Tecott LH Maricq AV Julius D Nervous system distribution of the serotonin 5-HT3 receptor mRNA Proc Natl Acad Sci USA 1993 90 1430 1434 10.1073/pnas.90.4.1430 8434003 PMC45887

[B34] Edagawa Y Saito H Abe K 5-HT1A receptor-mediated inhibition of long-term potentiation in rat visual cortex Eur J Pharmacol 1998 349 221 224 10.1016/S0014-2999(98)00286-6 9671101

[B35] Kojima T Matsumoto M Togashi H Tachibana K Kemmotsu O Yoshioka M Fluvoxamine suppresses the long-term potentiation in the hippocampal CA1 field of anesthetized rats: an effect mediated via 5-HT1A receptors Brain Res 2003 959 165 168 10.1016/S0006-8993(02)03756-3 12480170

[B36] Morales M Battenberg E de Lecea L Sanna PP Bloom FE Cellular and subcellular immunolocalization of the type 3 serotonin receptor in the rat central nervous system Brain Res Mol Brain Res 1996 36 251 260 8965645 10.1016/0169-328x(96)88406-3

[B37] Morales M Battenberg E de Lecea L Bloom FE The type 3 serotonin receptor is expressed in a subpopulation of GABAergic neurons in the rat neocortex and hippocampus Brain Res 1996 731 199 202 10.1016/0006-8993(96)00557-4 8883870

[B38] Yakel JL Jackson MB 5-HT3 receptors mediate rapid responses in cultured hippocampus and a clonal cell line Neuron 1988 1 615 621 10.1016/0896-6273(88)90111-0 3272181

[B39] Yakel JL Trussell LO Jackson MB Three serotonin responses in cultured mouse hippocampal and striatal neurons J Neurosci 1988 8 1273 1285 2965756 10.1523/JNEUROSCI.08-04-01273.1988 PMC6569259

[B40] Passani MB Pugliese AM Azzurrini M Corradetti R Effects of DAU 6215, a novel 5-hydroxytryptamine3 (5-HT3) antagonist on electrophysiological properties of the rat hippocampus Br J Pharmacol 1994 112 695 703 8075890 10.1111/j.1476-5381.1994.tb13132.x PMC1910391

[B41] Jones KA Surprenant A Single channel properties of the 5-HT3 subtype of serotonin receptor in primary cultures of rodent hippocampus Neurosci Lett 1994 174 133 136 10.1016/0304-3940(94)90004-3 7526284

[B42] Ropert N Guy N Serotonin facilitates GABAergic transmission in the CA1 region of rat hippocampus in vitro J Physiol 1991 441 121 136 1687746 10.1113/jphysiol.1991.sp018742 PMC1180189

[B43] Harrell AV Allan AM Improvements in hippocampal-dependent learning and decremental attention in 5-HT(3) receptor overexpressing mice Learn Mem 2003 10 410 419 10.1101/lm.56103 14557614 PMC218007

[B44] Ohashi S Matsumoto M Togashi H Ueno K Yoshioka M The serotonergic modulation of synaptic plasticity in the rat hippocampo-medial prefrontal cortex pathway Neuroscience Letters 2003 342 179 182 10.1016/S0304-3940(03)00293-3 12757894

[B45] Zhan H Tada T Nakazato F Tanaka Y Hongo K Spatial learning transiently disturbed by intraventricular administration of ouabain Neurol Res 2004 26 35 40 10.1179/016164104773026507 14977055

[B46] Rogers LJ Oettinger R Szer J Mark RF Separate chemical inhibitors of long-term and short-term memory: contrasting effects of cycloheximide, ouabain and ethacrynic acid on various learning tasks in chickens Proc R Soc Lond B Biol Sci 1977 196 171 195 10.1098/rspb.1977.0036 16264

[B47] Bell GA Gibbs ME Unilateral storage of monocular engram in day-old chick Brain Res 1977 124 263 270 10.1016/0006-8993(77)90884-8 843948

[B48] Glushchenko TS Izvarina NL [Na(+), K(+)-ATPase activity in the neurons and glial cells of the olfactory cortex of the rat brain in the dynamic development of long-term potentiation] Fiziol Zh Im I M Sechenova 1995 81 16 20 7581554

[B49] Mackler SA Kleyman TR Cha XY Regulation of the Na+/K(+)-ATPase pump in vitro after long-term exposure to cocaine: role of serotonin J Pharmacol Exp Ther 1998 285 835 843 9580634

[B50] Wang XQ Yu SP Novel regulation of Na, K-ATPase by Src tyrosine kinases in cortical neurons J Neurochem 2005 93 1515 1523 10.1111/j.1471-4159.2005.03147.x 15935067

[B51] Frazier CJ Buhler AV Weiner JL Dunwiddie TV Synaptic potentials mediated via alpha-bungarotoxin-sensitive nicotinic acetylcholine receptors in rat hippocampal interneurons J Neurosci 1998 18 8228 8235 9763468 10.1523/JNEUROSCI.18-20-08228.1998 PMC6792840

[B52] Frazier CJ Rollins YD Breese CR Leonard S Freedman R Dunwiddie TV Acetylcholine activates an alpha-bungarotoxin-sensitive nicotinic current in rat hippocampal interneurons, but not pyramidal cells J Neurosci 1998 18 1187 1195 9454829 10.1523/JNEUROSCI.18-04-01187.1998 PMC6792737

[B53] Nishikawa K MacIver MB Membrane and synaptic actions of halothane on rat hippocampal pyramidal neurons and inhibitory interneurons J Neurosci 2000 20 5915 5923 10934238 10.1523/JNEUROSCI.20-16-05915.2000 PMC6772580

[B54] Xiang Z Huguenard JR Prince DA GABAA receptor-mediated currents in interneurons and pyramidal cells of rat visual cortex J Physiol 1998 506 Pt 3 715 730 9503333 10.1111/j.1469-7793.1998.715bv.x PMC2230760

